# Effect of Surface Properties on the Photo-Induced Crawling Motion of Azobenzene Crystals on Glass Surfaces

**DOI:** 10.3389/fchem.2021.684767

**Published:** 2021-08-05

**Authors:** Yasuo Norikane, Masaru Hayashino, Mio Ohnuma, Koji Abe, Yoshihiro Kikkawa, Koichiro Saito, Kengo Manabe, Koji Miyake, Miki Nakano, Naoki Takada

**Affiliations:** ^1^ Research Institute for Advanced Electronics and Photonics, National Institute of Advanced Industrial Science and Technology (AIST), Tsukuba, Japan; ^2^ Department of Chemistry, Faculty of Pure and Applied Sciences, University of Tsukuba, Ibaraki, Japan; ^3^ Graduate School of Pure and Applied Sciences, University of Tsukuba, Ibaraki, Japan; ^4^ Advanced Manufacturing Research Institute, National Institute of Advanced Industrial Science and Technology (AIST), Tsukuba, Japan; ^5^ Research Institute for Energy Conservation, National Institute of Advanced Industrial Science and Technology (AIST), Tsukuba, Japan

**Keywords:** photochromism, crystal, azobenzene, actuation, phase transition

## Abstract

Photo-induced crawling motion of a crystal of 3,3′-dimethylazobenzene (DMAB) on a glass substrate having different surface properties was studied. When exposed to UV and visible lights simultaneously from different directions, crystals crawl continuously on a glass surface. On a hydrophilic surface, the crystals crawled faster than those on other surfaces but crystals showed spreading while they moved. On hydrophobic surfaces, on the other hand, the crystals showed little shape change and slower crawling motion. The contact angles of the liquid phase of DMAB on surface-modified glass substrates showed positive correlation with the water contact angles. The interaction of melted azobenzene with glass surfaces plays an important role for the crawling motion. We proposed models to explain the asymmetric condition that leads to the directional motion. Specifically by considering the penetration length of UV and visible light sources, it was successfully shown that the depth of light penetration is different at the position of a crystal. This creates a nonequilibrium condition where melting and crystallization are predominant in the same crystal.

## Introduction

The motion of artificial micro- to centimeter-sized objects, including crystals, photocatalyst particles, surface modified objects, functionalized particles, and polymers, driven by external stimuli has attracted significant attention (Xu et al., 2017; Kong et al., 2019; Dattler et al., 2020; Naumov et al., 2020). By selecting the materials, stimuli, and designing the system, diverse types of motion such as linear, rotary, and deformation can be achieved. Thus, it is of interest due to their potential application to micromotors, actuators, soft robots, and also their scientific viewpoint to reveal the mechanism. To date, various external stimuli such as electric fields, magnetic fields, pH, chemical gradient, and light have been used to induce the motion. Among those stimuli, light is very useful because it can be a powerful energy source and has spatiotemporal controllability. Light has been used to make objects move by using various types of mechanistic strategies such as photocatalytic reactions, photochromic reactions, and photothermal effects. Those generate locally nonequilibrium conditions and/or asymmetric fields in solution, within solids, or at interfaces.

Photochromic molecular switches such as azobenzenes and diarylethenes are molecules exhibiting isomerization between isomers by light (Goulet-Hanssens et al., 2020). The advantage of materials utilizing photochromic molecules is that their properties can be triggered noninvasively and reversibly. Additionally, the photochromic molecules change their molecular shape, which drastically changes molecular interactions, resulting in amplification of macroscopic properties such as mechanical motion. Azobenzene exhibits photoisomerization between the cis and trans isomers (Bandara and Burdette, 2012). Because of the difference of the absorption spectra of two isomers, trans -> cis and cis -> trans, isomerizations are generally dominant with UV and visible light, respectively. Various types of motion have been demonstrated in different states (polymer, crystal, amorphous, etc.) of azobenzene-containing materials. One of the most successful molecular systems in azobenzene-containing materials is photoactuators, especially in liquid crystal elastomers (Zhu et al., 2020; Chen et al., 2020). These polymers show contraction/expansion, bending, twisting, and oscillation by light and are expected to be used as 4D printing materials. Besides polymer materials, small molecules containing azobenzene are also known to exhibit motion by light. Single crystals of azobenzenes show bending motion, due to photoisomerization producing stress inside the crystal (Taniguchi et al., 2019). Amorphous solids of azobenzenes exhibit mass flow by a linearly polarized laser irradiation (Nakano and Suzuki, 2012; Suzuki and Nakano, 2012). Motion of an oil droplet was achieved by asymmetrical photoirradiation on a silica plate surface covered with azobenzene-containing molecules (Ichimura et al., 2000).

Recently, we found that crystals of simple azobenzenes, such as 3,3′-dimethylazobenzene (DMAB) and azobenzene, crawl on a glass surface by continuous photoirradiation of both UV and visible lights (365 and 465 nm) (Uchida et al., 2015). The motion is directional, and crystals move away from the UV light source. This crawling motion is continuous and can be achieved by a simple continuous irradiation using a light-emitting diode or Hg lamp as light sources in a fixed position. It is notable that no expensive apparatus such as a laser or positioning device is required. Nor is cumbersome temporal control necessary. It has been found that the motion of the crystals can be not only in a horizontal direction but also in a vertical one: they can climb the wall of glass surface. This crawling motion is very interesting due to its continuous behavior, even though these azobenzenes exhibit typical two state switching between trans and cis isomers. However, these crystals show phase transitions between crystal and liquid phases: melting and crystallization by trans -> cis and cis -> trans isomerization, respectively. This photochemically induced phase transition is considered as a key process for the crawling motion. In addition, we have successfully shown that the crawling motion can be induced by a single light source by using 4-(methylamino) azobenzene (Saito et al., 2019). Here, the liquid phase is readily converted to the crystal phase due to its short lifetime of the cis isomer.

Materials showing photochemically induced phase transitions between solid and liquid phases by the isomerization of azobenzene have been of interest because of their potential applications (Yamamoto et al., 2018; Norikane et al., 2020). This concept is simple; the trans isomer of azobenzene in the solid state melts by the irradiation of UV light due to the photoisomerization. Then, the cis isomer in the liquid state returns to the solid state via photochemical isomerization by visible light irradiation or thermal isomerization. After our report of the photoinduced crystal-to-isotropic phase transition in macrocyclic azobenzenes in 2011 (Norikane et al., 2011; Uchida et al., 2013), it has been shown that this phase transition is possible in different classes of molecules such as crystalline small molecules (Hoshino et al., 2014; Norikane et al., 2014; Baroncini et al., 2015; Ishiba et al., 2015; Norikane et al., 2016a; Norikane et al., 2016b; Hu et al., 2018; Yue et al., 2018), glassy branched middle molecules (Akiyama and Yoshida, 2012; Akiyama et al., 2014a; Akiyama et al., 2014b; Akiyama et al., 2014c), and viscous macromolecules (Akiyama et al., 2017; Zhou et al., 2017). Accordingly, the research area has been expanded to various kinds of smart materials such as reworkable adhesives (Akiyama and Yoshida, 2012; Akiyama et al., 2014a; Akiyama et al., 2014b; Akiyama et al., 2014c; Norikane et al., 2016a), photoresists (Norikane et al., 2014), gas storage (Baroncini et al., 2015), solar thermal fuels (Ishiba et al., 2015; Dong et al., 2018; Hu et al., 2019), self-healing (Zhou et al., 2017), switching enzymatic degradation of biolpolymers (Kikkawa et al., 2017), and swimming crystals (Norikane et al., 2016b).

The crawling motion of a crystal is intriguing because an object (crystal) is traveling on a solid surface (i.e., solid/air interface) under an ambient atmosphere. This is in contrast with numerous examples of stimuli responsive and self-propelling motion of small objects in solution or at solid/liquid interface (Dong et al., 2018). This crawling motion would be a candidate for a “carriage vehicle” to transport objects/chemicals on a solid surface without a flow channel. However, so far, the speed of the crawling motion is quite slow (up to 10 microns per min), and more importantly, the mechanism of the motion is not clear. Understanding the mechanism of the crawling motion is a prerequisite to establishing a system that can accelerate the speed and precisely control the shape and position of the crystals. It is presumed that the motion is driven by crystallization and melting at the front and rear edges of the crystal, respectively, *via* photochemical conversion between the crystal and liquid phases induced by the trans–cis isomerization of azobenzenes. Interaction between the azobenzene (either solid or liquid phase) and the glass surface may play an important role in the crawling motion. Thus, it motivates us to study this interaction.

In this work, we studied the crystal motion of DMAB on glass substrates with various surface wettability to reveal the effect of the interaction between the azobenzene and the glass surface. We used a hydrophilic glass, treated by KOH/EtOH solution, and two hydrophobic glasses, functionalized with 1,1,1,3,3,3-hexamethyldisilazane (HMDS) and 1H,1H,2H,2H-perfluorodecyltriethoxysilane. The crawling motion was observed by using a laser confocal microscope, and we found that the crawling motion was fastest on the hydrophilic surface although the shape of crystals became thinner during the motion. We proposed models to simulate the asymmetric condition that leads to the directional motion. It is shown that the depth of light penetration of UV and visible lights are different at the position of a crystal so there is a nonequilibrium condition where melting and crystallization are predominant in the same crystal.

## Experimental

3,3′-Dimethylazobenzene (DMAB) was purchased from Tokyo Chemical Industry Co., Ltd. and purified by silica gel column chromatography and subsequent recrystallization. Its purity was confirmed by NMR. Cover glasses (Matsunami Glass Ind., Ltd, square microscope cover glass No.1, 18 mm × 18 mm, thickness: 0.12–0.17 mm) were used as the glass substrates. Hydrophilic glass was prepared by dipping a cover glass in a saturated KOH/EtOH solution for 1 h and the glass was washed with EtOH. We prepared two hydrophobic surfaces (hydrophobic A and B). Hydrophobic A: by dipping a cover glass in an HMDS (1,1,1,3,3,3-hexamethyldisilazane) without dilution for overnight, then washing with ethanol. Hydrophobic B: a cover glass cleaned by a UV ozone cleaner (Nippon Laser & Electronics Lab.). Then, by spin coating (3,000 rpm for 45 s) onto the cover glass with a solution of 1H,1H,2H,2H-perfluorodecyltriethoxysilane (1 vol% solution in ethanol), followed by curing (100°C, 10 min). Surface contact angles of water and DMAB were measured by using a contact angle meter (Kyowa Interface Science Co., Ltd., DMe-210 and DM-500).

To obtain similarly sized crystals on a substrate without disturbing the substrate surface, we used sieves. A glass substrate was placed under a stainless steel sieve (45 and 20 μm) and crystals of DMAB were passed through these sieves (Supplementary Figure 1). This method is different from the one in the previous report where we recrystallized from the melted state on a glass substrate (Uchida et al., 2015).

**FIGURE 1:**
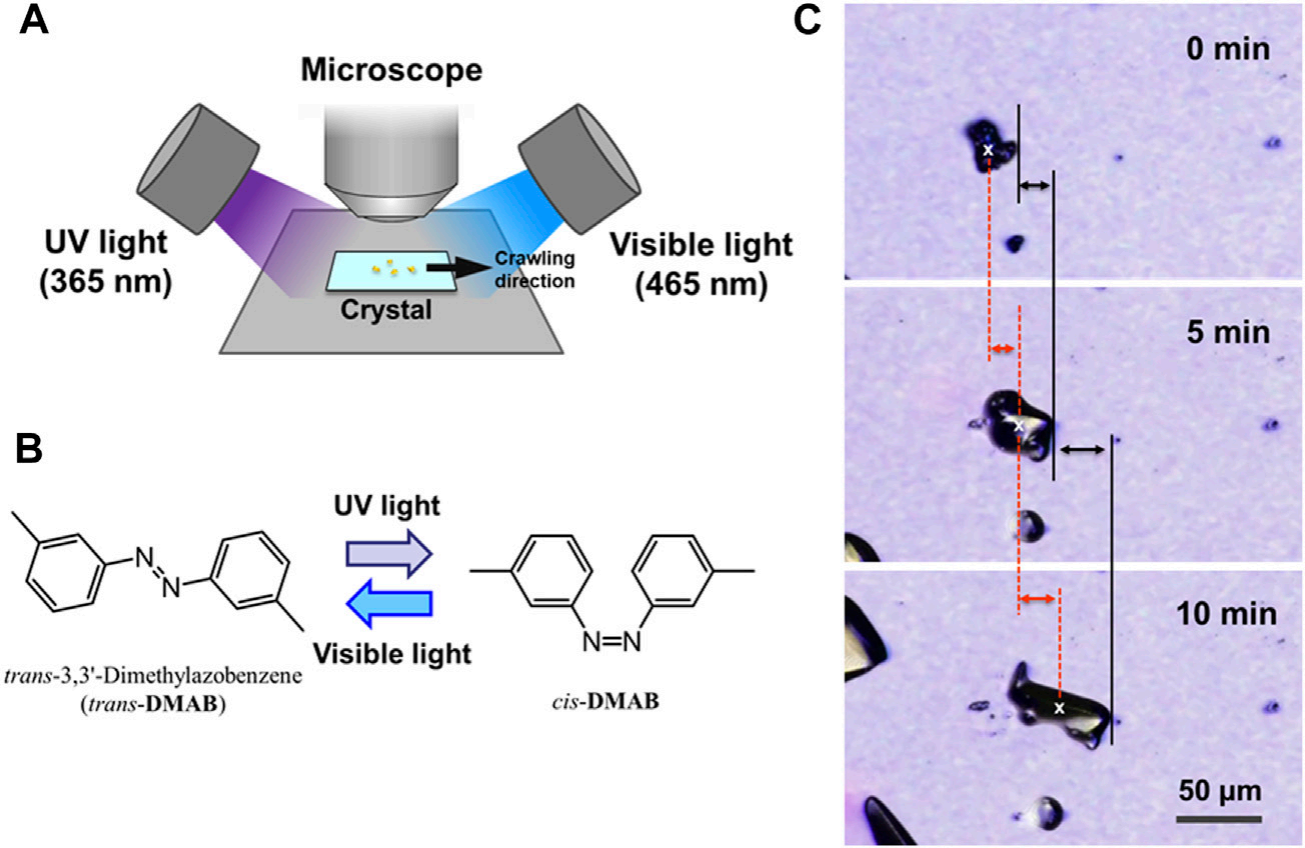
**(A)** Schematic diagram of the experimental setup for the observation of the crawling crystals. UV (365 nm) and visible (465 nm) light were irradiated from opposite directions. The angle between the direction of light and the plane of glass was 30° for each light source. The photomicrograph was taken every minute. **(B)** Molecular structure of DMAB and its photoisomerization scheme on irradiation with UV and visible light. **(C)** Examples of microscope images of DMAB crystals during the crawling motion, displaying the definition of the distance of the motion. The moving distance of the crystal motion was defined by measuring the positions of the front edge (black lines) and the center of the crystal (red dashed lines). The front edge was defined at the end at the right side of the crystal. The center of the crystal (x marks) was defined by an image analysis software.

For the motion of the crystals on a substrate, photoirradiation was carried out with 365 and 465 nm using the experimental setup shown in Figure 1A under ambient temperature. LED’s were used for 365 nm (CCS Inc., HLV-24UV365-4WNRBTNJ) and 465 nm (CCS Inc., HLV2-22BL-3W). The light intensity was monitored using a Newport 1917-R optical power meter with an 818-ST-UV photodetector. The intensity of the 365 and 465 nm in this study were 200 and 50 mW cm^−2^, respectively. The motion of the crystals was observed using a Keyence VK-X100 laser confocal microscope with a laser wavelength of 658 nm. Microphotographs were taken every 1 min. The velocity of the crystal motion was measured based on the front edge and the center of the crystals as shown in Figure 1C. The position of the center of a crystal was defined as a centroid of each image.

## Results and Discussion

The crystal samples examined here were prepared by scattering the crystals of DMAB on a glass substrate through a mesh as shown in Supplementary Figure 1. This method is different from the one in our previous report where small pieces of crystals of DMAB, formed by cooling the melted state sandwiched between two cover glasses, were obtained by peeling the cover glasses (Uchida et al., 2015). In the previous method, it was difficult to control the size of the crystals. In addition, the surface of the glass substrate is not in a “fresh” condition in the previous method because the whole surface of glass plates had contacted the DMAB crystal before detaching them. In order to reduce the surface contamination, it is desirable to place crystals, having suitable size for the crawling motion, directly on a glass substrate. We have attempted to deposit and grow small crystals on a fresh glass substrate by sublimating DMAB, but it did not work. The deposited compound formed small liquid droplets instead of forming crystals. The deposited droplets were in the supercooled state and were stable for hours. They slowly evaporated even at ambient condition at room temperature without forming crystals and finally disappeared from the substrate. We have also tried to deposit a concentrated solution on a glass substrate by a micro manipulator or an inkjet printer. However we obtained only liquid droplets and did not form microcrystals. In addition, we have applied a reprecipitation method by dropping DMAB solutions into water (poor solvent), but only oil droplets were formed in the solution. Alternatively, we then adopted the method of scattering through the mesh. Although the crystal shape cannot be controlled, this method is currently the most efficient way to place a lot of microcrystals of DMAB on a large area of solid surface without disturbing the freshly prepared surface. Actually, we have prepared the crystals on a glass surface without surface treatment, and we confirmed the motion of the crystals. The irradiation condition and the behavior were similar to those reported in the previous report (Uchida et al., 2015).

In this study, the motion of the crystals was analyzed by defining the position of the crystal at the front edge and the center as shown in Figure 1C. The crystal motion involves the change in the crystal shape. Especially when the crystals deform into thinner shape, the area projected on a plane becomes larger. In this case, the position of the front edge moves much faster than the center when the crystal is spreading to the moving direction. Therefore by evaluating the position of both the front edge and the center, we can discuss the degree of spreading of the crystals.

**FIGURE 2:**
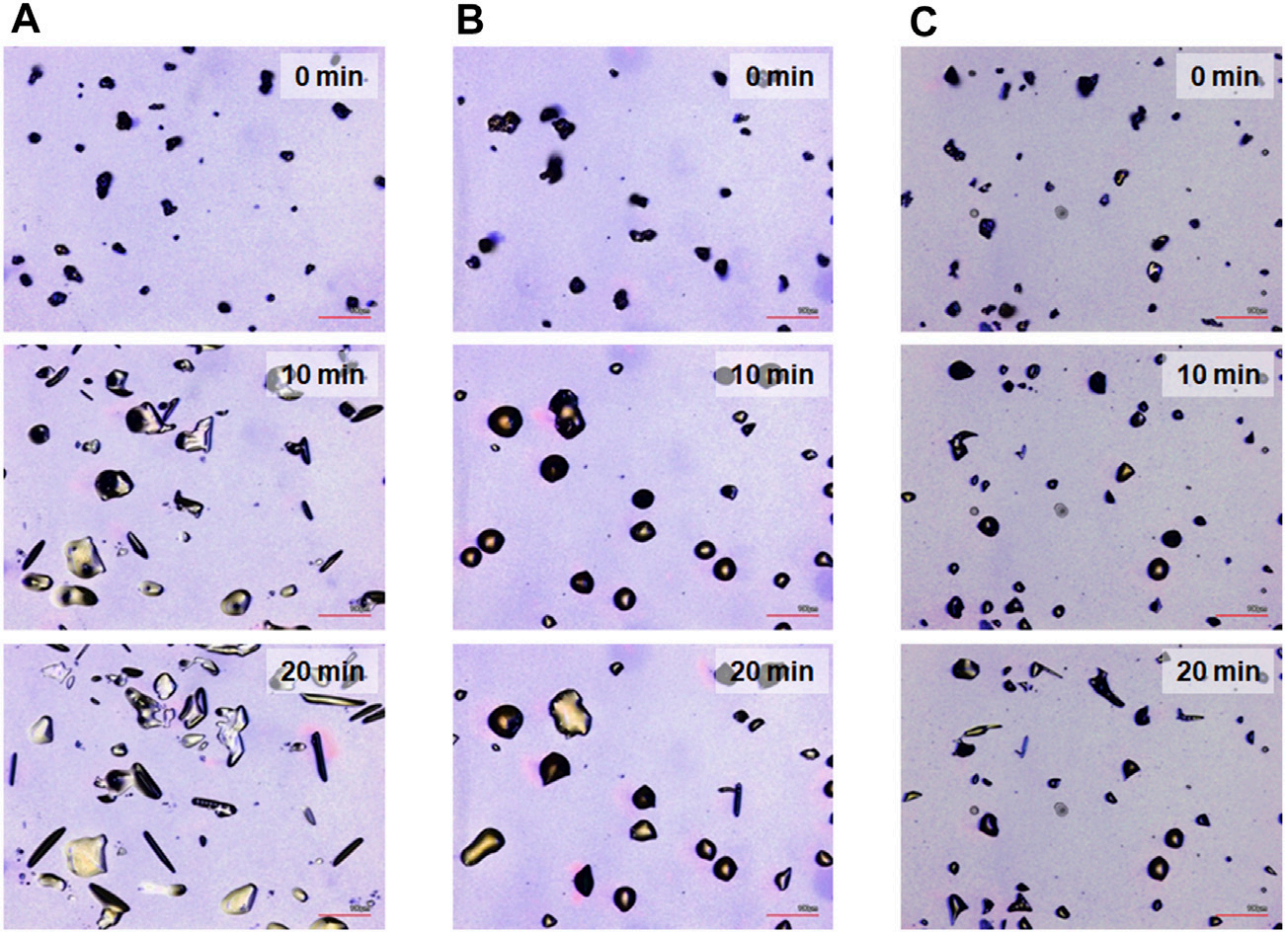
Photomicrographs for the crawling motion of DMAB crystals on three different glass surfaces [**(A)** hydrophilic, **(B)** hydrophobic A, and **(C)** hydrophobic B] after irradiation for t = 0, 10, and 20 min, respectively. Scale bar: 100 μm.

**FIGURE 3:**
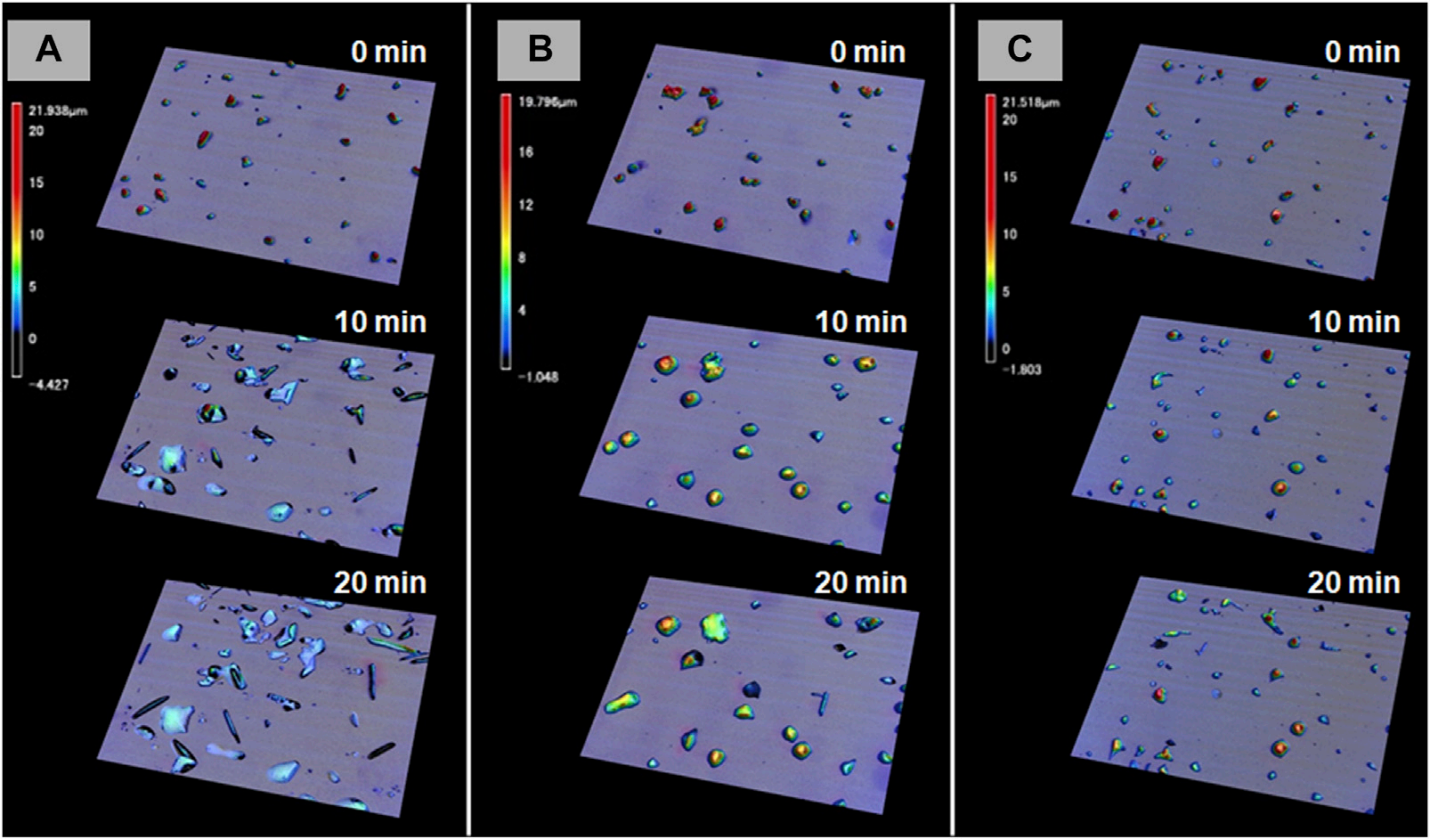
3D photomicrographs observed by a confocal laser microscope on three different glass surfaces [**(A)** hydrophilic, **(B)** hydrophobic A, and **(C)** hydrophobic B] after irradiation for t = 0, 10, and 20 min, respectively.

**FIGURE 4:**
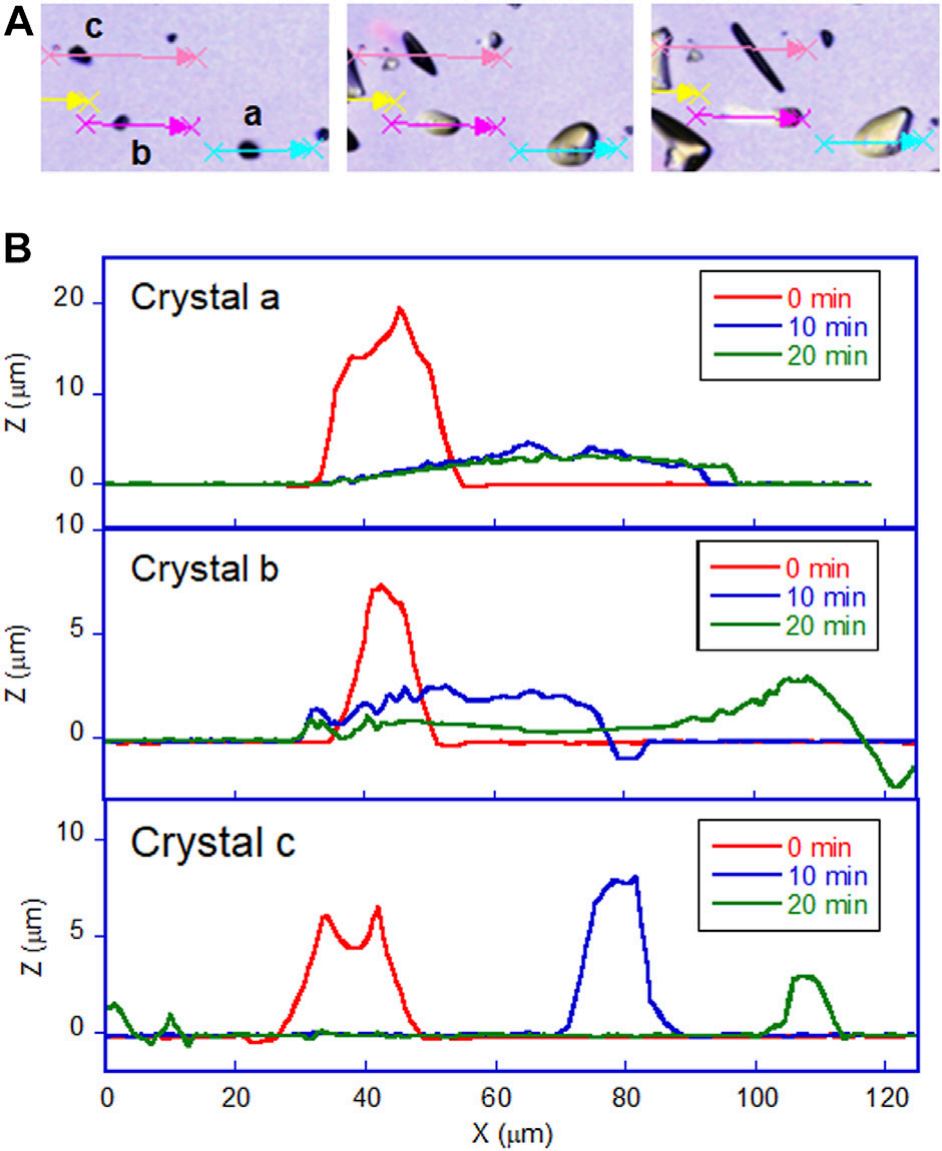
Profile of DMAB crystals on hydrophilic glass surface observed by laser confocal microscope. **(A)** Photomicrographs of crystals on hydrophilic glass at t = 0, 10, and 20 min of irradiation. Letters a, b, and c indicate the crystals selected to analyze those profiles along with the arrows. **(B)** The profile of each crystal with irradiation time at t = 0, 10, and 20 min.

The most prominent motion was observed on the hydrophilic glass. Figure 2A shows the photomicrographs of the DMAB crystals on the hydrophilic glass. The movie of the motion can be found in Supplementary Video 1. Originally we observed the particles with the size of ca. 10–40 μm and they were in the crystal phase confirmed by the polarizing optical microscope. The simultaneous UV and visible light irradiation changed those crystals into a “spread” shape within a few minutes and the crystals started crawling toward the visible light source. The spreading was observed as shown in the 3D images (Figure 3A) observed by a laser confocal microscope. By looking at how the crystals change their shape over time, there are mainly two types of crystal morphology. One is spreading equally to the *x* and *y* direction forming a flattened shape. In this case, the crystals become thinner during the motion but their centers definitely move. The profile analyses of the crystal by a laser confocal microscope revealed that a crystal of the original height of ca. 20 μm (with similar x and y dimensions) changed its shape to have a height of 4 μm and diameter of ca. 50 μm (“crystal a” in Figure 4). Notably, the profile of the crystal is asymmetric, in other words the front side of the crystal has a steeper edge than its rear end. Another case is a spreading toward two opposite directions of the crystal forming a rod-like shape (“crystal c” in Figure 4). Interestingly, this rod-like shape crystal crawls regardless of the relative orientation of the rod to the direction of the light source. The rod-like crystal became longer with time. The profile of both edges of the long side of a crystal is symmetrical and steep. It is in contrast with the first case that has an asymmetrical profile. The occurrence of these two types of the spreading phenomena is probably due to the crystal orientation at the original state. The original crystal orientation determines how the crystal spreads because the original crystal acts as a “seed” and the crawling motion is a combination of melting and growth of the crystal. Since the crystal is attached on the glass surface, it is difficult to turn the crystal orientation. It is a rather interesting fact that the crawling motion was observed in either case. The speed of the motion seems to depend on the orientation of the crystal; therefore the motion can be precisely controlled by using originally oriented crystals. The velocity of the crystals reported here is based on the average of each speed of crystal in different orientations. The velocity on the hydrophilic glass was thus 2.5 μm min^−1^ when the position of the center of crystal was averaged, while the front edge moves at 3.3 μm min^−1^ (Table 1). This velocity was more than four times faster than that observed on an untreated glass surface (0.6 μm min^−1^ at the center of the crystal).

**TABLE 1:** Static contact angles of water and DMAB (at 60°C), and velocity of DMAB crystals at their center and front edge on different surfaces.

Substrate	Water contact angle (degree)	DMAB contact angle at 60°C (degree)	Velocity of center of crystal (μm min^−1^)	Velocity of front edge of crystal (μm min^−1^)
Hydrophilic	5	5	2.5	3.3
Glass	53	31	0.6	1.3
Hydrophobic A	73	39	0.7	1.5
Hydrophobic B	98	60	0.3	1.4

**FIGURE 5:**
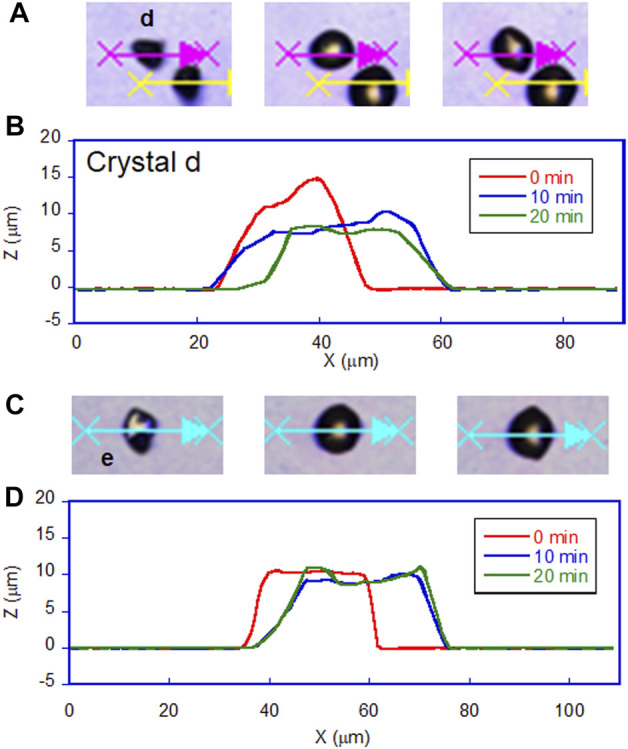
Profile of DMAB crystals on hydrophobic glass surfaces observed by laser confocal microscope. **(A)** Photomicrographs of crystals on hydrophobic A glass at t = 0, 10, and 20 min of irradiation. Letter d indicates the crystal selected to analyze its profile along with the arrow. **(B)** The profile of the crystal d with irradiation time at t = 0, 10, and 20 min. **(C)** Photomicrographs of crystals on hydrophobic B glass at t = 0, 10, and 20 min of irradiation. Letter e indicates the crystal selected to analyze its profile along with the arrow. **(D)** The profile of the crystal e with irradiation time at t = 0, 10, and 20 min.

On hydrophobic surfaces, the crawling motion was also observed but the velocity of the motion was slower than that on the hydrophilic surface. Figure 2B shows the snapshots of the motion on the hydrophobic A surface. The time-lapse movie is shown in Supplementary Video 2. The average velocity on this surface was 0.7 and 1.5 μm min^−1^ at the center and the front edge of the crystal, respectively (Table 1). By the simultaneous irradiation of the UV and visible light, the crystal changed to “spread” shapes, but this time the degree of the spreading was less than the crystals on the hydrophilic surface (Figure 2B). For example, a crystal with its original diameter of ca. 30 and 15 μm in height deformed and the diameter and the height became ca. 40 and 10 μm, respectively, after 10 min of irradiation (Figures 5A,B). Interestingly, this crystal exhibited a motion that is like an inchworm movement. During the initial 10 min of irradiation the front edge moved forward, and the shrinking of the rear edge was observed at the subsequent 10 min. This is presumably due to the effect of pinning of the liquid phase of the DMAB by this hydrophobic surface. Pinning behavior was also observed on this surface when we attempted to measure dynamic contact angle and sliding angles by using liquid droplets of DMAB at elevated temperature (at 60°C). Not all crystals exhibited such inchworm-like crawling behavior but we found it frequently on this surface. On the hydrophobic B surface, on the other hand, the velocity of the crawling motion was 0.3 and 1.4 μm min^−1^ at the center and the front edge of the crystal, respectively (Table 1). Those are slightly slower than that observed on the hydrophobic A surface. On this surface, the crystals did not show noticeable spreading by the photoirradiation (Figures 2C, 3C and Supplementary Video 3). In addition, the height of the crystals did not change as shown in Figures 5C,D.

**FIGURE 6:**
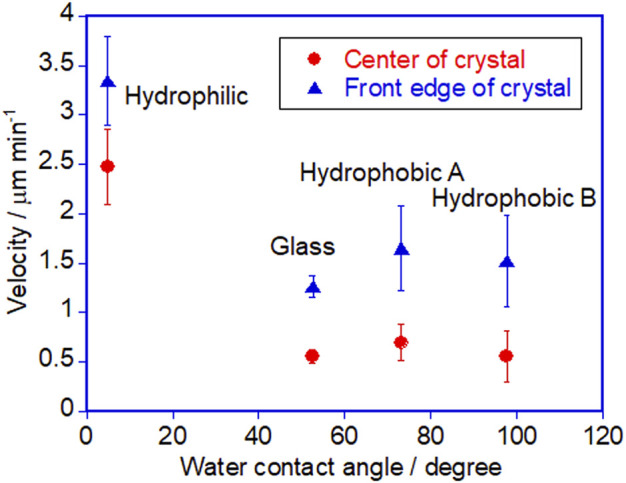
Plot of the velocity of the crystal against the water contact angle of different surfaces. Error bars represent standard error.

Figure 6 shows the plot of the velocity of the crawling motion against the water contact angle of the surface. As can be clearly seen from the plot, the speed of the motion increases on a hydrophilic surface. The average velocity on a hydrophilic surface was 2.5 μm min^−1^ analyzed at the center of crystals. Indeed it is more than four times faster than that on an untreated glass surface (0.6 μm min^−1^). On the other hand, the velocity values on the two hydrophobic surfaces and untreated glass are similar. It is interesting that there is no significant effect on the velocity from the surface wettability in these glass surfaces, although there was an observed difference in crystal shape between the hydrophobic surfaces as shown in Figures 3B,C. On the hydrophobic A surface, crystals exhibit more spreading than those on the hydrophobic B surface. Those observations indicate that the difference in spreading of crystals is caused by the surface wettability, but this does not affect the speed of the crawling motion, probably because the extent of spreading is too small.

In the above discussion, the surface wettability is evaluated based on the water contact angle. However, instead of water contact angle, we have to clarify the interaction of DMAB to the glass surface. Thus, verification is necessary for whether the water contact angle is a reliable parameter for assessing the effect of the surface qualitatively. We measured the contact angles of DMAB droplets. Since DMAB crystallizes at room temperature, the measurements were conducted at 60°C, above the melting point of DMAB (53°C and 43–46°C for trans (Yamamoto et al., 1971; Uchida et al., 2015) and cis (Badger et al., 1953; Uchida et al., 2015), respectively). It was found that there is an almost linear positive correlation between the DMAB contact angle and the water contact angle (Table 1 and Supplementary Figure 2). This result shows that the water contact angle can be used as a parameter to discuss the effect of wettability of DMAB. By using the droplet of DMAB, the contact angle hysteresis was also measured. On hydrophilic glass, the advancing angle and receding angle were 10.2° and 6.3°, respectively. On hydrophobic B glass, their values were 65.5° and 48.5°, respectively, for the hydrophobic B surface. On the other hand, we could not determine the dynamic contact angles for the hydrophobic A surface due to the pinning of the droplet.

Here, let us discuss the mechanism of the crawling motion, by pointing out the possible factors that affect the motion. As mentioned in the introduction, it is presumed that the crawling motion is driven by crystallization and melting at the front and rear edges of the crystal, respectively, via photochemical conversion between the crystal and liquid phases induced by the trans–cis isomerization of azobenzenes. However, the photoirradiation is carried out uniformly on the whole crystal, which means that both the front edge and the rear edge of the crystal are irradiated. First, let us consider the gradient of light intensity created by the distance from the light source. When looking at one crystal, the difference of the distance from the light source between the front edge and the rear edge is ca. 20 μm, that is, the size of the crystal. In our experimental condition, the distance from the crystal to the light source is about 3 cm. Since the light intensity decreases by an inverse-square law, the intensity decrease was calculated to be only ca. 0.13% from one end to the other. Therefore the light intensity gradient by the distance of the light source is not the main factor for the crawling motion.

Second, it should be noted that there might be an effect of a shadow created by the crystal. Since the light is from 30° above the plane of the glass surface, a shadow can be formed when the slope of the crystal edge is more than 30°. Figures 4, 5 clearly show that most of the initial state of crystals has the shape of a steep enough edge for creating a shadow. In these crystals, one edge of the crystal is irradiated selectively by one light source and the other edge is irradiated by another light source. It seems to be a very efficient way for melting and crystallizing the crystal at the opposite edge selectively. This model was our initial hypothetical model for explaining the crawling motion. However, this model cannot explain two experimental observations. 1) On hydrophobic surfaces the crystals kept their shape that may have a shadow but the velocity is quite low and the motion seemed inefficient. 2) On hydrophilic surfaces, the crystals change their shape to become very thin such that the shadow becomes small or disappears, but the velocity of the thin crystals is faster than those on other surfaces. Therefore, creating the shadow is not always effective for the crawling motion, and instead, surface wettability seems to have a more strong impact on the velocity.

**FIGURE 7:**
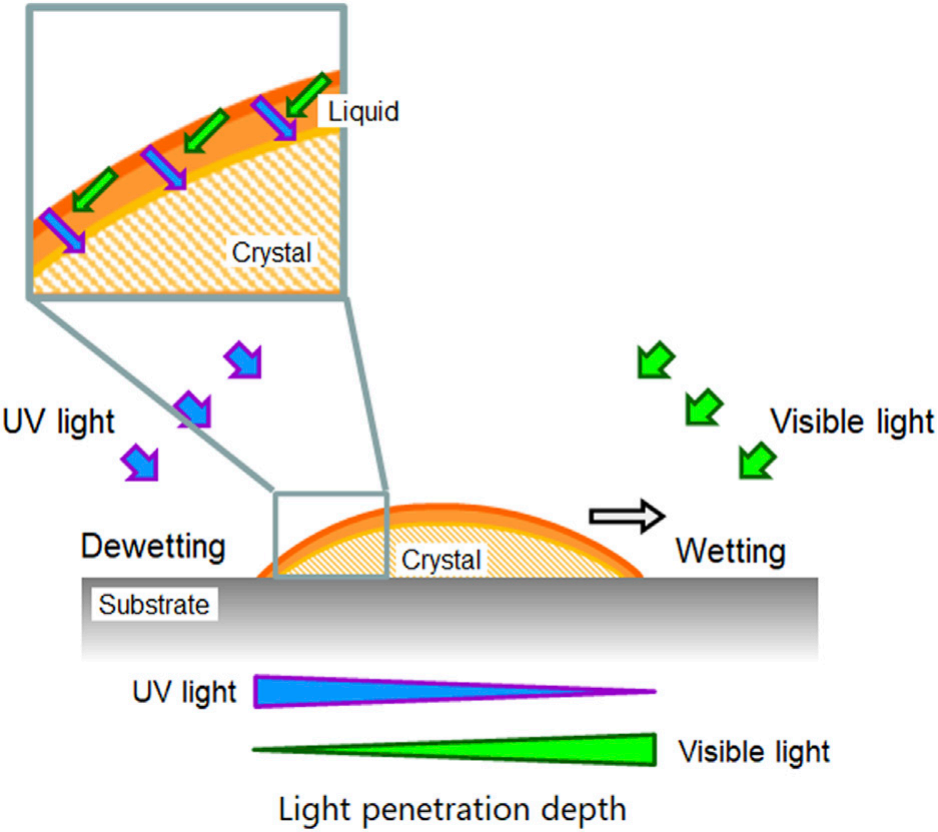
A simple schematic diagram showing the penetration length of light. See the text for the detail.

Next, there still remains the question of why those very thin crystals still crawl. It should explain the reason for the directional motion without the “shadow hypothesis”. How do two light sources create an asymmetric environment for directional motion? To get closer to the answer of this question, we would like to discuss according to the path length and the penetration of each light on the surface of a crystal. A simple model is shown in Figure 7 with a crystal that is thin enough to have no shadow. Let us consider the crystal surface at the rear side of the crystal. Because the crystal surface has a slope and the surface is facing to the UV light source, the angle of the UV light to the surface is greater than that of the visible light. Here we can expect that UV light would reach deeper inside the crystal, if the penetration length (shown as arrows in Figure 7) of each light is identical, which will be assumed for the moment simplify this explanation (penetration depth will be discussed later). If UV light reaches deeper into the crystal, the surface of the crystal melts to the liquid phase due to the trans - > cis photoisomerization. Thus, in this side of the crystal, the melting can occur more than the crystallization. At the front edge of the crystal, on the other hand, we can expect that the crystallization takes place more than the melting because the visible light reaches deeper in the crystal. Overall, the melting and crystallization are continuously taking place in the same crystal which can be regarded as a nonequilibrium condition that creates an asymmetric environment. Actually, at the interface of the crystal and liquid of DMAB, adsorption and desorption of DMAB molecules (mainly trans isomer) is taking place and there is a local equilibrium at each position of a crystal. The melted azobenzene liquid shows wetting and spread on the surface isotropically if there is no parameter that controls the direction of the wetting. The melted liquid portion of the crystal is actually pulled by the growing part of crystal at the front edge. Due to the affinity between the substrate surface and the liquid phase, the dewetting process at the rear edge of the crystal is slow so the shape of the crystal becomes thinner during the crawling motion. For now there is no clear evidence that the spreading of the liquid state is the only factor for the crawling motion. We think the growing of the crystal is a more important factor. The growing speed of the crystal can be influenced by the surface energy. Further study is required to distinguish two factors such as the spreading of the droplet and the growing of the crystal.

The simple model described above is based on a very rough assumption that the penetration length of two wavelengths of light is the same. However, as a matter of course, those penetration lengths are different because the extinction coefficient (*ε)* of azobenzene is different at different wavelengths. Here let us discuss the depth of light penetration. According to the Lambert–Beer law, the light intensity (*I*) at certain optical path length (*L*) in the sample is expressed for UV and visible lights as follows:

$$I^{uv}=I_{0}^{uv}10^{-\varepsilon^{uv}L^{uv}C}.$$ (1)

$$I^{vis}=I_{0}^{vis}10^{-\varepsilon^{vis}L^{vis}C}.$$ (2)

Here, *I*_0_ and *C* are the initial intensity of light and the concentration, respectively. By using Eqs 1, 2, we have simulated the decay of the light intensities of each wavelength inside the crystal as shown in Supplementary Figure 4. In this simulation, for simplicity, the angle of light is fixed at 90° to the plane of the crystal, and reflection and scattering are omitted. The value of $\varepsilon^{uv}$ and $\varepsilon^{vis}$ are fixed as constant values of 5,000 and 500 M^−1^cm^−1^, respectively, and the density value (Uchida et al., 2015) of 1.17 g mol^−1^ from crystal data was used for the calculation of the concentration. In the graph, a steep decay of the UV light is due to the relatively large $\varepsilon^{uv}$. On the other hand, it should be noted that the visible light can penetrate far deeper inside the crystal. Importantly, the intensity of the visible light $\left(I^{vis}\right)$ becomes predominant over that of the UV light $\left(I^{uv}\right)$ in the crystal deeper than ca. 0.24 μm. This result clearly matches with the experimental results that a crystal does not melt completely but melting is taking place only at the periphery of the crystal when two lights are irradiated simultaneously.

**FIGURE 8:**
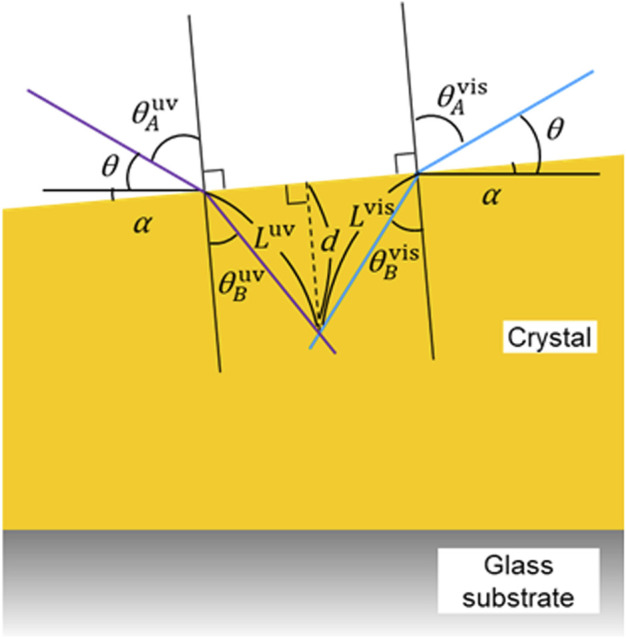
A schematic diagram showing the model for simulating the angle, path length, and penetration depth of light at the rear edge of a crystal. See the text for the details.

With those things in hand, estimation of the light intensity was carried out by considering our experimental conditions including oblique light directions, refractive index, refraction angle, and slope angle of the crystal. According to the Snell’s law, the incoming light on to the surface of crystal is refracted as shown in Figure 8 and Eqs 3, 4:

$$\frac{\sin\theta_{A}^{uv}}{\sin\theta_{B}^{uv}}=n_{AB}.$$ (3)

$$\frac{\sin\theta_{A}^{vis}}{\sin\theta_{B}^{vis}}=n_{AB}.$$ (4)

Here, $\theta_{A}^{\;uv}$ and $\theta_{A}^{\;vis}$ are the angle of UV and visible light from the normal of the crystal surface, respectively, $\theta_{B}^{\;uv}$ and $\theta_{B}^{\;vis}$ are the refracted angle of UV and visible light from the normal of the crystal surface, respectively, and $n_{AB}$ is the relative refractive index of the crystal. Thus, Eqs 3, 4 are written as follows:

$$\theta_{B}^{\;uv}=\sin^{-1}\left(\frac{\sin\theta_{A}^{\;uv}}{n_{AB}}\right).$$ (5)

$$\theta_{B}^{\;vis}=\sin^{-1}\left(\frac{\sin\theta_{A}^{\;vis}}{n_{AB}}\right).$$ (6)

As shown in Figure 8, the angle of the light source to the plane of the glass and the slope of the crystal surface to the plane of the glass are defined as *θ* and *α*, respectively. The relationship between $\theta_{A}^{\;uv}$, $\theta_{A}^{\;vis}$, *θ,* and *α* are the following:

$$\theta_{A}^{\;uv}=90-\left(\theta +\alpha \right).$$ (7)

$$\theta_{A}^{\;vis}=90-\left(\theta -\alpha \right).$$ (8)

By using these relationships, Eqs 5, 6 can therefore be written as the following:

$$\theta_{B}^{uv}=\sin^{-1}\left\{\frac{\sin\left(90-\theta +\alpha \right)}{n_{AB}}\right\}.$$ (9)

$$\theta_{B}^{vis}=\sin^{-1}\left\{\frac{\sin\left(90-\theta -\alpha \right)}{n_{AB}}\right\}.$$ (10)

On the other hand, by considering the optical path lengths of each light ($L^{uv}$ and $L^{vis}$) at certain depth (d) from the surface of the crystal, relation of those values are as follows:

$$L^{uv}=\frac{d}{\cos\theta_{B}^{uv}},$$ (11)

$$L^{vis}=\frac{d}{\cos\theta_{B}^{vis}},$$ (12)

and the ratio of the two optical path length is as follows:

$$\frac{L^{vis}}{L^{uv}}=\frac{\cos\theta_{B}^{uv}}{\cos\theta_{B}^{vis}}.$$ (13)

According to Eqs 9, 10, 13, the ratio of the optical path length is a function of *θ*, *α,* and $n_{AB}$. By using the value of $n_{AB}=1.61$ in the literature (Auwers and Heimke, 1928) and *θ* = 30° at our experimental condition, the ratio is plotted as shown in Supplementary Figure 5. It clearly shows that the optical path length of the visible light becomes greater than that of the UV light when the slope of the crystal becomes steeper.

Finally, let us consider the light intensity. By combining Eqs 1, 2, 9–12, we can estimate the light intensity of each light ($I^{uv}$ and $I^{vis}$) at certain depth as follows:

$$I^{uv}=I_{0}^{uv}10^{-\varepsilon^{uv}\frac{d}{\cos\theta_{B}^{uv}}C}=I_{0}^{uv}10^{-\varepsilon^{uv}dC/\cos\left[\sin^{-1}\left\{\frac{\sin\;\left(90-\theta +\alpha \right)}{n_{AB}}\right\}\right]}.$$ (14)

$$I^{vis}=I_{0}^{vis}10^{-\varepsilon^{vis}\frac{d}{\cos\theta_{B}^{vis}}C}=I_{0}^{vis}10^{-\varepsilon^{vis}dC/\cos\left[\sin^{-1}\left\{\frac{\sin\;\left(90-\theta -\alpha \right)}{n_{AB}}\right\}\right]}.$$ (15)

By using Eqs 14, 15, we have simulated the light intensities when the slope *α* = 5, 15, and 25°. The simulated results are shown in Supplementary Figures 6A, 7A, 8A. It should be noted that those curves are different. When *α* increases, the simulated curve of the UV light intensity becomes relatively gentle decay, while the curve of the visible light intensity becomes sharper decay. As a result, the intersection between two curves (UV and visible light) shifts to the deeper *d* value (*d*_*i*_) from 0.21 to 0.23 μm. This result agrees well to the simple “arrow” model (Figure 7), that UV light can reach deeper inside the crystal when the crystal surface is facing to the UV light source.

Similar to the above discussion, we simulated the light intensity at the front edge of the crystal in the condition with the slope *α* = 5, 15, and 25° as shown in Supplementary Figures 6B, 7B, 8B, respectively (the definition of the angles is shown in Supplementary Figure 9). At this edge of the crystal, the simulated curve of the UV light intensity becomes steeper decay when *α* increases. The intersection of two curves shifts to the shallower *d*_*i*_ from 0.20 to 0.18 μm. By using the results of the simulation, we can discuss the nonequilibrium condition created in a crystal on the surface. Let us consider a crystal having a symmetric shape such that the slope angles of the rear and the front edge are identical. When *α* = 5°, the *d*_*i*_ of the rear edge is 0.21 μm where that of the front edge is 0.20 μm. As *α* increases, the difference of *d*_*i*_ at two edge becomes more prominent. The *d*_*i*_ of the rear and the front edge are 0.23 and 0.18 μm, respectively, when *α* = 25°. These results indicate that the degree of melting is different at the different edges of a crystal and it may produce an asymmetric condition in a crystal.

Those simulations shown above are still primitive approximations. We used fixed value for the molar extinction coefficients ($\varepsilon^{uv}$and $\varepsilon^{vis}$) and refractive index ($n_{AB}$). However, the actual absorbance of the sample changes due to the photoisomerization of azobenzene and the isomer distribution varies by the position and the depth of a crystal. For example, the absorption of UV light decreases with trans to cis photoisomerization. In addition, the absorption changes when the crystal melts due to the change in intermolecular interactions. The values of refractive index may differ between crystal and liquid phases as well as trans and cis isomers. Thus, the light penetration depth and the angle of propagation should be more dynamic so that the degree of the penetration changes when the crystal changes its shape due to the change of the isomer ratio. However, even by using primitive assumption, the simulation qualitatively revealed the difference in the light intensity of UV and visible light in the crystal. These results clearly prove the existence of the nonequilibrium condition that leads to the crawling motion.

Finally, we would like to mention about the surface roughness of the glass surface. We have attempted to see the effect of the roughness of the glass surface, however the effect seemed to be very small compared to the effect of the wettability. In addition, the surface of the glass we used was difficult to control the roughness with reproducibility to conduct a series of experiments to assess the effect of the roughness. Alternatively, we are currently investigating the effect of the roughness by using other substrates with controlled roughness. We will report the results elsewhere.

## Conclusion

To understand the mechanism of the photo-induced crawling motion of the crystal of DMAB, the effect of surface property was investigated by using glass substrate with different surface treatments. On a hydrophilic surface, the crystals crawled faster, but the spreading of crystals was observed during the motion. On hydrophobic surfaces, the crystals showed little shape change and slower crawling motion. The contact angles of the liquid phase of DMAB on surface modified glass substrates showed positive correlation with the water contact angles. Thus, the liquefied DMAB spread more on hydrophilic surface. Therefore, the interaction of melted azobenzene with glass surfaces plays an important role for the speed of crawling motion. We developed models that those explain the asymmetric condition that leads to the directional motion. A shadow model was simple, but only this model could not explain the fact that thinner crystals still show the crawling motion on the hydrophilic surface. By considering the penetration length of UV and visible light sources, it was successfully shown that the depth of light penetration is different at the positions of a crystal so this creates a nonequilibrium condition where melting and crystallization are predominant in the same crystal. The model shown here is yet primitive, but it will contribute to revealing the mechanism of the crawling motion in more detail by considering dynamic change in parameters. In addition, by establishing more precise simulation models, it may provide the way to design novel crystal systems for improved crawling motion.

## Data Availability

The original contributions presented in the study are included in the article/Supplementary Material, further inquiries can be directed to the corresponding author.

## References

[B1] Akiyama H. Fukata T. Yamashita A. Yoshida M. Kihara H. (2017). Reworkable Adhesives Composed of Photoresponsive Azobenzene Polymer for Glass Substrates. J. Adhes. 93, 823–830. 10.1080/00218464.2016.1219255

[B2] Akiyama H. Kanazawa S. Okuyama Y. Yoshida M. Kihara H. Nagai H. (2014). Photochemically Reversible Liquefaction and Solidification of Multiazobenzene Sugar-Alcohol Derivatives and Application to Reworkable Adhesives. ACS Appl. Mater. Inter. 6, 7933–7941. 10.1021/am501227y 24818772

[B3] Akiyama H. Kanazawa S. Yoshida M. Kihara H. Nagai H. Norikane Y. (2014). Photochemical Liquid-Solid Transitions in Multi-Dye Compounds. Mol. Crystals Liquid Crystals 604, 64–70. 10.1080/15421406.2014.967743

[B4] Akiyama H. Yoshida M. Kihara H. Norikane Y. Azumi R. (2014). Organic Photofunctional Materials Composed of Azobenzene Derivatives: Liquid-Solid Phase Transition in Multi Azobenzene Compounds with Partially Substituted Structures. J. Photopol. Sci. Technol. 27, 301–305. 10.2494/photopolymer.27.301

[B5] Akiyama H. Yoshida M. (2012). Photochemically Reversible Liquefaction and Solidification of Single Compounds Based on a Sugar Alcohol Scaffold with Multi Azo-Arms. Adv. Mater. 24, 2353–2356. 10.1002/adma.201104880 22488711

[B6] Auwers K. v. Heimke P. (1928). Spektrochemische Beobachtungen an Azoverbindungen. Chem. Ber. 61, 1030–1036.

[B7] Badger G. M. Buttery R. G. Lewis G. E. (1953). 440 Aromatic Azo-Compounds. Part I. Oxidation of Cis- and Trans-azobenzene. J. Chem. Soc., 2143–2147. 10.1039/jr9530002143

[B8] Bandara H. M. D. Burdette S. C. (2012). Photoisomerization in Different Classes of Azobenzene. Chem. Soc. Rev. 41, 1809–1825. 10.1039/c1cs15179g 22008710

[B9] Baroncini M. d'Agostino S. Bergamini G. Ceroni P. Comotti A. Sozzani P. (2015). Photoinduced Reversible Switching of Porosity in Molecular Crystals Based on star-shaped Azobenzene Tetramers. Nat. Chem 7, 634–640. 10.1038/nchem.2304 26201739

[B10] Chen M. Liang S. Liu C. Liu Y. Wu S. (2020). Reconfigurable and Recyclable Photoactuators Based on Azobenzene-Containing Polymers. Front. Chem. 8, 706. 10.3389/fchem.2020.00706 32974276 PMC7471039

[B11] Dattler D. Fuks G. Heiser J. Moulin E. Perrot A. Yao X. (2020). Design of Collective Motions from Synthetic Molecular Switches, Rotors, and Motors. Chem. Rev. 120, 310–433. 10.1021/acs.chemrev.9b00288 31869214

[B12] Dong L. Feng Y. Wang L. Feng W. (2018). Azobenzene-based Solar thermal Fuels: Design, Properties, and Applications. Chem. Soc. Rev. 47, 7339–7368. 10.1039/c8cs00470f 30168543

[B13] Goulet‐Hanssens A. Eisenreich F. Hecht S. (2020). Enlightening Materials with Photoswitches. Adv. Mater. 32, 1905966. 10.1002/adma.201905966 31975456

[B14] Hoshino M. Uchida E. Norikane Y. Azumi R. Nozawa S. Tomita A. (2014). Crystal Melting by Light: X-ray Crystal Structure Analysis of an Azo Crystal Showing Photoinduced Crystal-Melt Transition. J. Am. Chem. Soc. 136, 9158–9164. 10.1021/ja503652c 24918317

[B15] Hu J. Huang S. Yu M. Yu H. (2019). Flexible Solar Thermal Fuel Devices: Composites of Fabric and a Photoliquefiable Azobenzene Derivative. Adv. Energ. Mater. 9, 1901363. 10.1002/aenm.201901363

[B16] Hu J. Li X. Ni Y. Ma S. Yu H. (2018). A Programmable and Biomimetic Photo-Actuator: a Composite of a Photo-Liquefiable Azobenzene Derivative and Commercial Plastic Film. J. Mater. Chem. C 6, 10815–10821. 10.1039/c8tc03693d

[B17] Ichimura K. Oh S.-K. Nakagawa M. (2000). Light-Driven Motion of Liquids on a Photoresponsive Surface. Science 288, 1624–1626. 10.1126/science.288.5471.1624 10834837

[B18] Ishiba K. Morikawa M.-A. Chikara C. Yamada T. Iwase K. Kawakita M. (2015). Photoliquefiable Ionic Crystals: A Phase Crossover Approach for Photon Energy Storage Materials with Functional Multiplicity. Angew. Chem. Int. Ed. 54, 1532–1536. 10.1002/anie.201410184 25483773

[B19] Kikkawa Y. Tanaka S. Norikane Y. (2017). Photo-triggered Enzymatic Degradation of Biodegradable Polymers. RSC Adv. 7, 55720–55724. 10.1039/c7ra10598c

[B20] Kong L. Mayorga‐Martinez C. C. Guan J. Pumera M. (2019). Photocatalytic Micromotors Activated by UV to Visible Light for Environmental Remediation, Micropumps, Reversible Assembly, Transportation, and Biomimicry. Small 16, 1903179. 10.1002/smll.201903179 31402632

[B21] Nakano H. Suzuki M. (2012). Photoinduced Mass Flow of Photochromic Molecular Materials. J. Mater. Chem. 22, 3702–3704. 10.1039/c2jm16517a

[B22] Naumov P. Karothu D. P. Ahmed E. Catalano L. Commins P. Mahmoud Halabi J. (2020). The Rise of the Dynamic Crystals. J. Am. Chem. Soc. 142, 13256–13272. 10.1021/jacs.0c05440 32559073

[B23] Norikane Y. Hirai Y. Yoshida M. (2011). Photoinduced Isothermal Phase Transitions of Liquid-Crystalline Macrocyclic Azobenzenes. Chem. Commun. 47, 1770–1772. 10.1039/c0cc04052e 21125115

[B24] Norikane Y. Saito K. Yue Y. (2020). “Crawling and Bending Motions of Azobenzene Derivatives Based on Photoresponsive Solid-Liquid Phase Transition System,” in Photosynergetic Responses in Molecules and Molecular Aggregates. Editors Miyasaka H. Matsuda K. Abe J. Kawai T. (Singapore: Springer), 465–478. 10.1007/978-981-15-5451-3_27

[B25] Norikane Y. Tanaka S. Uchida E. (2016). Azobenzene Crystals Swim on Water Surface Triggered by Light. Cryst. Eng. Comm. 18, 7225–7228. 10.1039/c6ce00738d

[B26] Norikane Y. Uchida E. Tanaka S. Fujiwara K. Koyama E. Azumi R. (2014). Photoinduced Crystal-to-Liquid Phase Transitions of Azobenzene Derivatives and Their Application in Photolithography Processes through a Solid-Liquid Patterning. Org. Lett. 16, 5012–5015. 10.1021/ol502223u 25216186

[B27] Norikane Y. Uchida E. Tanaka S. Fujiwara K. Nagai H. Akiyama H. (2016). Photoinduced Phase Transitions in Rod-Shaped Azobenzene with Different Alkyl Chain Length. J. Photopol. Sci. Technol. 29, 149–157. 10.2494/photopolymer.29.149

[B28] Saito K. Ohnuma M. Norikane Y. (2019). Negative Phototactic Behaviour of Crystals on a Glass Surface. Chem. Commun. 55, 9303–9306. 10.1039/c9cc03826d 31309947

[B29] Suzuki M. Nakano H. (2012). Moving Fragments of Photochromic Molecular Glass of 4-[Bis(9,9-Dimethylfluoren-2-Yl)amino]-4^|^rsquo;-Cyanoazobenzene. J. Photopol. Sci. Technol. 25, 159–160. 10.2494/photopolymer.25.159

[B30] Taniguchi T. Asahi T. Koshima H. (2019). Photomechanical Azobenzene Crystals. Crystals 9, 437. 10.3390/cryst9090437

[B31] Uchida E. Azumi R. Norikane Y. (2015). Light-Induced Crawling of Crystals on a Glass Surface. Nat. Commun. 6, 7310. 10.1038/ncomms8310 26084483 PMC4557305

[B32] Uchida E. Sakaki K. Nakamura Y. Azumi R. Hirai Y. Akiyama H. (2013). Control of the Orientation and Photoinduced Phase Transitions of Macrocyclic Azobenzene. Chem. Eur. J. 19, 17391–17397. 10.1002/chem.201302674 24318266

[B33] Xu L. Mou F. Gong H. Luo M. Guan J. (2017). Light-driven Micro/nanomotors: from Fundamentals to Applications. Chem. Soc. Rev. 46, 6905–6926. 10.1039/c7cs00516d 28949354

[B34] Yamamoto S. Nishimura N. Hasegawa S. (1971). Steric Effects in Azo Compounds. The Electric Dipole Moments and the Absorption Spectra of Azobenzene Derivatives. Bcsj 44, 2018–2025. 10.1246/bcsj.44.2018

[B35] Yamamoto T. Norikane Y. Akiyama H. (2018). Photochemical Liquefaction and Softening in Molecular Materials, Polymers, and Related Compounds. Polym. J. 50, 551–562. 10.1038/s41428-018-0064-4

[B36] Yue Y. Norikane Y. Azumi R. Koyama E. (2018). Light-Induced Mechanical Response in Crosslinked Liquid-Crystalline Polymers With Photoswitchable Glass Transition Temperatures. Nat. Commun. 9, 3234. 10.1038/s41467-018-05744-x 30104602 PMC6089925

[B37] Zhou H. Xue C. Weis P. Suzuki Y. Huang S. Koynov K. (2017). Photoswitching of Glass Transition Temperatures of Azobenzene-Containing Polymers Induces Reversible Solid-To-Liquid Transitions. Nat. Chem 9, 145–151. 10.1038/nchem.2625 28282043

[B38] Zhu C. Lu Y. Sun J. Yu Y. (2020). Dynamic Interfacial Regulation by Photodeformable Azobenzene-Containing Liquid Crystal Polymer Micro/Nanostructures. Langmuir 36, 6611–6625. 10.1021/acs.langmuir.0c00582 32449856

