# Supplementary material for: Effect of Surface Properties on the Photo-Induced Crawling Motion of Azobenzene Crystals on Glass Surfaces
Source: Front Chem. 2021 Aug 5;9:684767. doi: 10.3389/fchem.2021.684767 (PMC8374144; doi:10.3389/fchem.2021.684767)
Supplement: Supplementary file 2 [file DataSheet1.PDF]

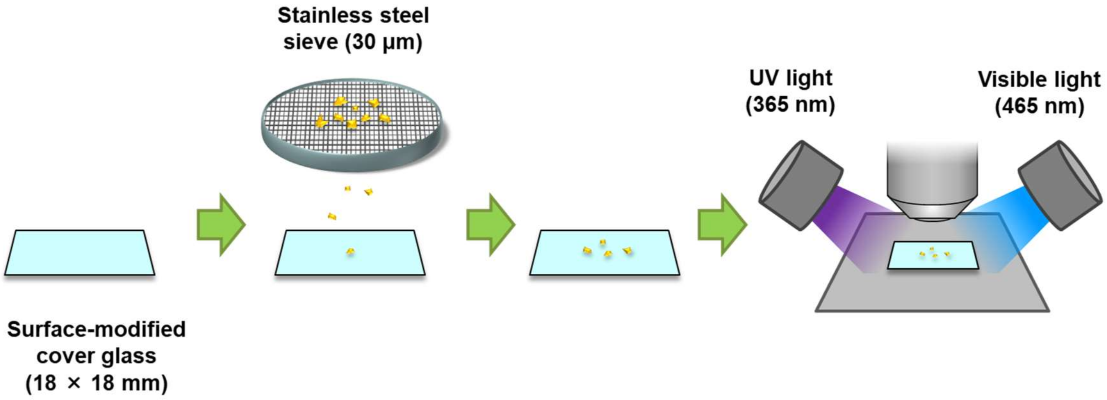

FIGURE S1 Schematic diagram of the preparation of crystal sample on a glass. Crystal powder of DMAB was passed through a stainless sieve and placed on a glass. The sample was then irradiated and observed by a microscope. To determine the center of the crystal an image analysis software (ImageJ) was used[1,2].

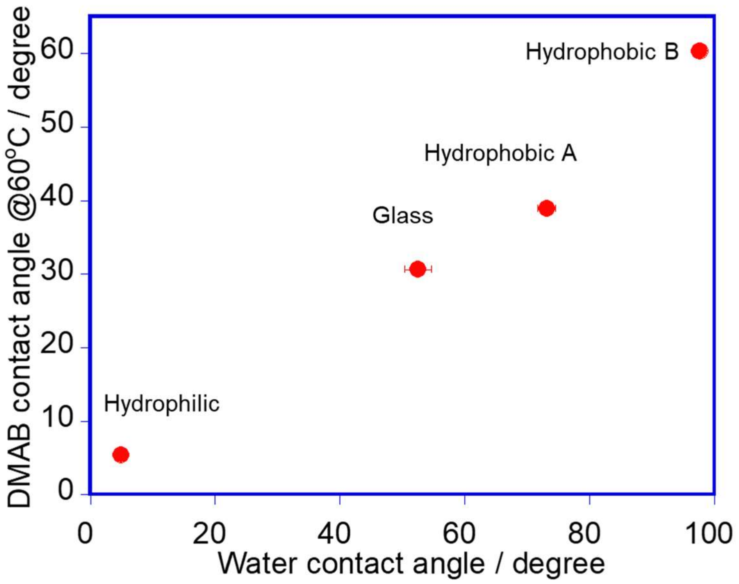

FIGURE S2 Plot of the correlation between the DMAB contact angle and the water contact angle on different glass surfaces. Error bars represent standard error.

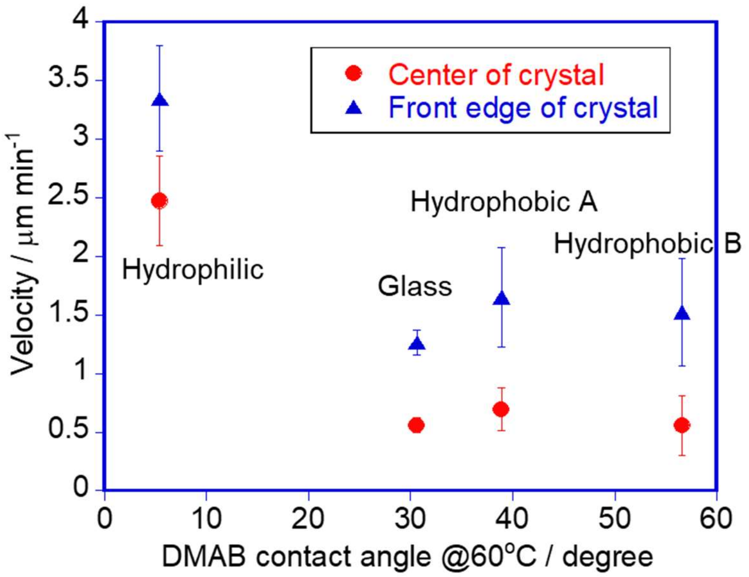

FIGURE S3 Plot of the velocity of the crystal against the DMAB contact angle of different surfaces. Error bars represent standard error.

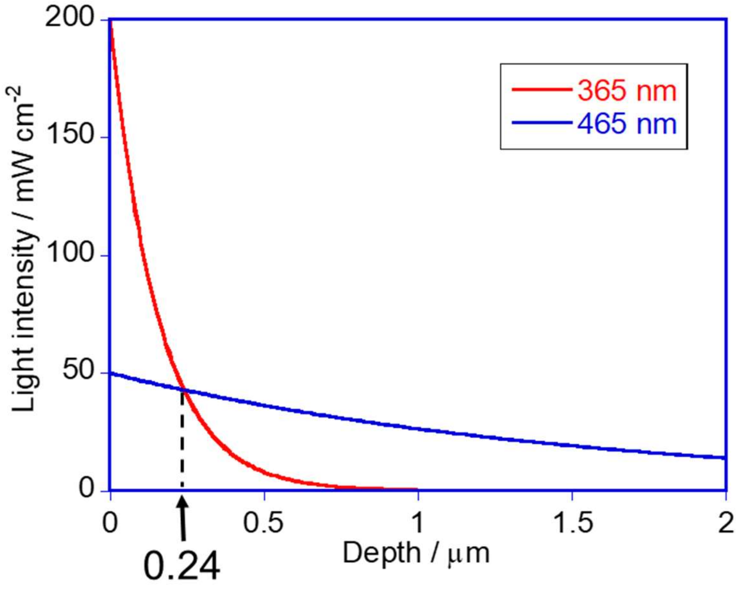

FIGURE S4 Simulated decay of the light intensities of each wavelength inside crystal when the angle of light is fixed at 90° to the plane of the crystal.

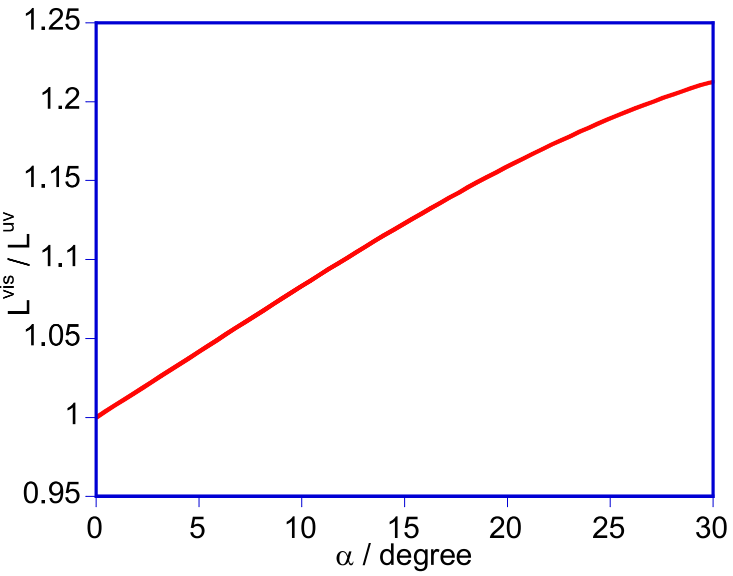

FIGURE S5 The ratio of the path length (L^{vis}/L^{UV}) against the slope of the crystal.

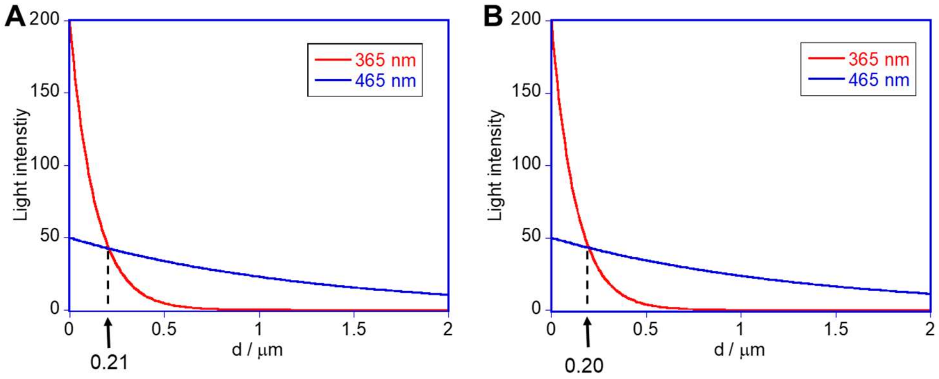

FIGURE S6 Simulated decay of the light intensities of each wavelength inside crystal at the rear edge (A) and the front edge (B) when the angle of slope (α) is 5°.

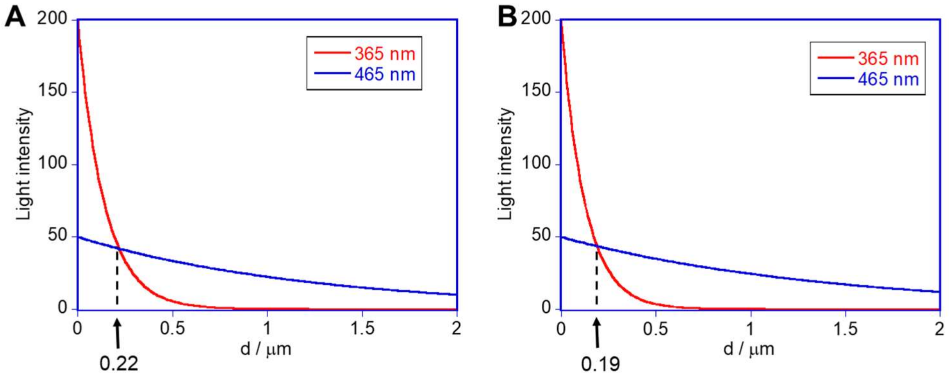

FIGURE S7 Simulated decay of the light intensities of each wavelength inside crystal at the rear edge (A) and the front edge (B) when the angle of slope (α) is 15°.

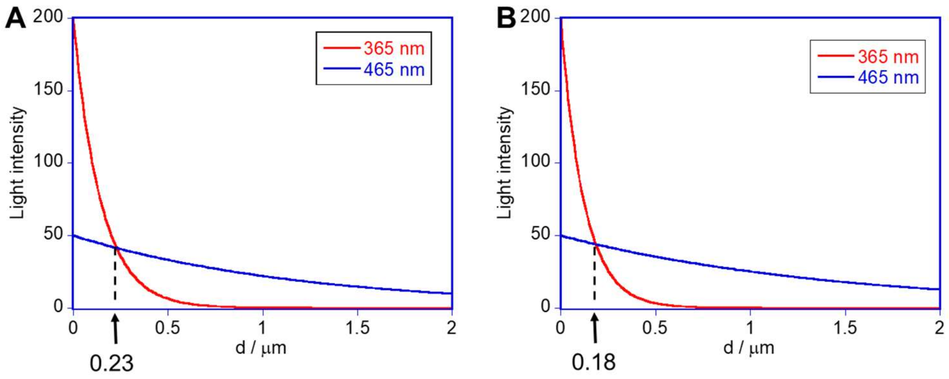

FIGURE S8 Simulated decay of the light intensities of each wavelength inside crystal at the rear edge (A) and the front edge (B) when the angle of slope (α) is 25°.

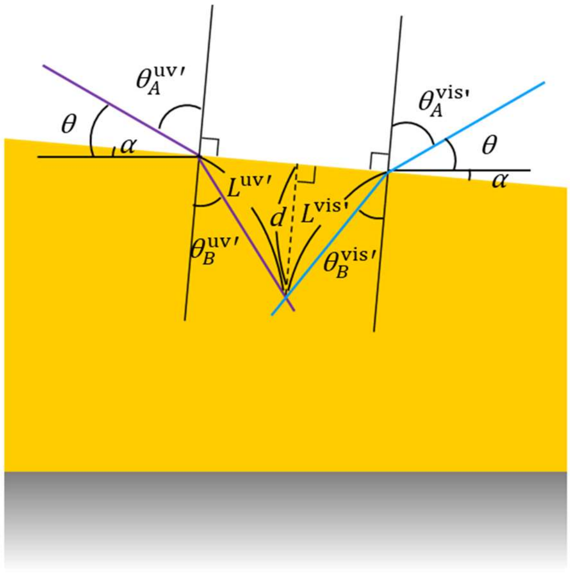

FIGURE S9 A schematic diagram showing the model for simulating the angle, path length, and penetration depth of light at the front edge of a crystal.

Reference

1) Rasband, W.S., ImageJ, U. S. National Institutes of Health, Bethesda, Maryland, USA, https://imagej.nih.gov/ij/, 1997-2019.
2) Schneider, C.A., Rasband, W.S., Eliceiri, K.W. "NIH Image to ImageJ: 25 years of image analysis". Nature Methods 9, 671-675, 2012.
